# Effects of different physical activity interventions on executive function in older adults with dementia: a systematic review and network meta-analysis

**DOI:** 10.3389/fneur.2025.1643957

**Published:** 2025-10-02

**Authors:** Ying Zhu, Shuang Li, Zixian Xiao, Hongyu Wang, Xiaolin Zhang, Qianqian Huang, Rui Yin, Kelei Guo, Dong Li

**Affiliations:** ^1^School of Physical Education and Health, Guangxi Normal University, Guilin, China; ^2^School of Physical Education and Health, Zhaoqing University, Zhaoqing, China

**Keywords:** older adults, dementia, executive function, physical activity, network meta-analysis

## Abstract

**Background:**

The executive function impairments in older adults with dementia have profound effects on their daily lives, families, and society. Physical activity has gained recognition as a complementary treatment for enhancing executive function in elderly individuals with dementia. Numerous studies have explored the correlation between physical activity and the enhancement of executive functions in dementia. Nevertheless, there remains a lack of comprehensive, systematic evidence that addresses crucial issues in identifying the most effective physical activity interventions. The primary objective of this study is to evaluate and rank different physical activity intervention strategies, offering valuable therapeutic guidance for improving executive function in older adults with dementia.

**Methods:**

We conducted a systematic search across four databases—PubMed, the Cochrane Library, Embase, and Web of Science—to locate randomized controlled trials (RCTs) evaluating the effect of physical activity interventions on executive function in individuals diagnosed with dementia. The search covered the period from January 2000 to May 2025. Two independent researchers performed the literature screening, data extraction, and quality assessment. A network meta-analysis was conducted using Stata 15.1.

**Results:**

A total of 24 studies were included in the analysis. The results indicated that resistance exercise ranked first in enhancing executive function among older adults with dementia, with the highest Surface Under the Cumulative Ranking Curve (SUCRA) (89.2%) and PrBest (59.9%) values. This was followed by mind–body exercises, with SUCRA (71.4%) and PrBest (18.9%), and aerobic exercise ranked third with SUCRA (60.0%) and PrBest (4.2%).

**Conclusion:**

Our findings suggest that both resistance exercise and mind–body exercise are likely more effective in enhancing executive function in older adults with dementia. Future studies should aim to design personalized physical activity programs that consider genetic factors, phenotypic characteristics, and cognitive baselines. Observing the long-term therapeutic effect and investigating the Intervention Mechanism, such as the regulation of brain-derived neurotrophic factor and the connectivity of the prefrontal cortex, to further optimize strategies for enhancing executive function in older adults with dementia.

**Systematic review registration:**

https://www.crd.york.ac.uk/prospero/, identifier CRD420251040158.

## 1 Introduction

Dementia is a clinical condition characterized by a gradual deterioration in cognitive functions such as memory, language, and executive function, with Alzheimer’s disease being the most common neurodegenerative form (1). According to the global disease burden study published in The Lancet, the number of dementia patients worldwide is increasing exponentially, with projections indicating an increase from 57.4 million cases in 2019 to 152.8 million by 2050 (2). More concerning is the 38% increase in mortality rates among older adults over the past decade (3), a trend expected to persist. Dementia has emerged as one of the primary causes of death among older adults (4, 5), placing significant strain on individuals, families, and healthcare infrastructures.

Executive function, a fundamental cognitive process primarily governed by the prefrontal cortex, is essential for managing behavioral control as individuals age (6). Through key modules such as planning, decision-making, short-term memory management, impulse control, and cognitive flexibility, executive function supports the adaptive responses of older adults to environmental changes (7). Neuroimaging studies indicate that the functional connectivity of the dorsolateral prefrontal cortex and anterior cingulate gyrus declines with age (6, 8), reducing attention allocation, task sequencing, and cortico-basal ganglia circuit coordination (9). In daily life, the decline in executive function significantly affects the quality of life of older adults, directly influencing their ability to plan, make decisions, and manage emotions, thereby determining their adaptability and task performance in social and work environments (10, 11). Furthermore, deficits in executive function are strongly linked to critical functional outcomes in dementia. Specifically, EF impairments predict difficulties in performing instrumental activities of daily living (IADLs) such as managing finances and medications, as well as basic activities of daily living (ADLs) like dressing and bathing. They are also associated with an increased risk of falls, poorer adherence to treatment plans, and impaired decision-making capacity, which collectively contribute to increased caregiver burden and reduced quality of life for both patients and their families. Therefore, targeting executive function through physical activity may yield broad benefits that extend beyond cognitive test scores to encompass these vital aspects of everyday functioning and well-being. When executive function is impaired, older adults may experience slow decision-making, distractibility, and difficulty managing complex tasks (12, 13), further compromising their independence and quality of life.

Treatment approaches for executive dysfunction in older adults with dementia primarily include pharmacological and non-pharmacological interventions, both of which exhibit clear limitations in mechanism and efficacy. Pharmacologically, cholinesterase inhibitors (e.g., donepezil) improve prefrontal information integration by inhibiting acetylcholine breakdown (14, 15), while NMDA receptor antagonists (e.g., memantine) mitigate neuronal damage via modulation of glutamate excitotoxicity (16). However, these treatments are constrained by significant individual variation in response, influenced by genetics, blood–brain barrier function, and disease stage (17). Moreover, existing drugs only alleviate symptoms without halting neurodegeneration, and long-term use may cause adverse effects such as gastrointestinal discomfort and cardiovascular issues, challenging adherence and safety (18). Non-pharmacologically, cognitive training and behavioral therapy foster functional compensation through sustained cognitive stimulation (19, 20). Yet, observable benefits generally require prolonged regular participation (21). For patients with moderate to severe impairment, gains in complex executive functions are often limited, and outcomes are moderated by baseline cognition, motivation, and social support, restricting broader applicability (22). In contrast, physical activity enhances core executive functions such as attention and planning by promoting neurogenesis, improving cerebral blood flow, and boosting overall health. It represents a safe and sustainable intervention that can delay decline and improve daily functioning across all disease stages (23).

Physical activity, as a non-pharmacological intervention, demonstrates significant potential for improving executive function in older adults with dementia. Empirical studies indicate that aerobic exercise, tai chi, and cycling can not only alleviate dementia symptoms but also effectively enhance executive abilities (24–27). Mechanisms such as modulating neurochemical levels, increasing cerebral blood flow, improving sleep quality, and strengthening psychological resilience contribute to improvements in attention, planning, and problem-solving (28, 29). Compared to pharmacological treatments, physical activity is associated with fewer side effects, greater cost-effectiveness, and better adherence, enhancing its relevance in modern healthcare. Tailoring exercise type and intensity to individual health status and preferences can further optimize outcomes (30). Such interventions can be applied either independently or in combination with drugs and psychotherapy to improve overall efficacy. In summary, physical activity offers a safe, effective, and practicable non-pharmacological strategy, particularly valuable for medication-averse patients or those emphasizing quality of life (31).

Prior meta-analyses have explored the relationship between physical activity and executive function in individuals with dementia (32). However, a gap remains in directly comparing and ranking the efficacy of different physical activity interventions for improving executive function, specifically in individuals with clinically diagnosed dementia. This gap is particularly pronounced and clinically significant in low- and middle-income countries (LMICs)—regions experiencing an alarming doubling of dementia incidence every 5 years (33), yet where high-quality, context-specific research evidence for non-pharmacological interventions remains scarce. This is especially critical for resource-limited settings that urgently require cost-effective non-pharmacological strategies. To address this gap, this study conducted a systematic review and network meta-analysis focused specifically on comparing different types of physical activity interventions targeting executive function in this population. To our knowledge, this is the first NMA focused specifically on types of physical activity targeting executive function in clinically diagnosed dementia. The dual objectives were to evaluate the comparative effectiveness of these interventions and to establish a ranked hierarchy of treatments for this specific clinical context. This research provides evidence-based guidance for clinicians to optimize exercise prescription programs aimed at enhancing executive function in older adults with dementia.

## 2 Methods

### 2.1 Protocol and registration

This study followed the 2020 guidelines of the Preferred Reporting Items for Systematic Reviews and Meta-Analyses (PRISMA), ensuring compliance with standards related to literature selection, data management, statistical analysis, and reporting of findings. Additionally, the study has been registered in the PROSPERO database (CRD 420251040158).

### 2.2 Data sources and search strategy

A thorough literature search was performed to examine the link between physical activity and executive function in dementia, utilizing four electronic databases: PubMed, Cochrane Library, Embase, and Web of Science. The search period covered data from January 1, 2000, to May 4, 2025, for each database. Based on the PICOS (Population, Intervention, Comparison, Outcome, Study Design) framework, the search terms included “Physical Activity” or “Activities, Physical” or “Aerobic activity” or “Recreation activities” or “Free-time activities” or “Leisure-time physical activity” or “Dementia” or “Neurocognitive Disorders” or “Senile Dementia” or “Alzheimer’s Disease” or “Vascular Dementia” or “Lewy Body Dementia” or “Older Adults” or “Aged” or “aging population” or “elderly” or “executive function” or “cognitive flexibility” or “Randomized controlled trial” or “randomized” For detailed search strategies, please consult Appendices B, B1.

### 2.3 Study selection

Following the implementation of the search strategy outlined above, authors YZ and SL independently carried out the initial literature screening. This preliminary step involved examining the titles and abstracts of the retrieved articles to identify studies that could be potentially relevant. Full-text assessments were then performed on the articles deemed to be more pertinent. Studies meeting the predefined inclusion criteria were ultimately selected for statistical analysis. In instances where discrepancies arose, the research team engaged in discussions to resolve any disagreements and reach a consensus.

### 2.4 Inclusion and exclusion criteria

This systematic review, guided by the PICOS framework, defined specific criteria for the selection, inclusion, and exclusion of studies.

The criteria for including literature were as follows:

1. The study population consisted of older adults with a confirmed diagnosis of dementia, aged ≥ 50 years.
2. Interventions involved different forms of exercise or physical activity.
3. The research provided data on executive function outcomes in dementia patients before and after the intervention.
4. Only RCTs were considered eligible for inclusion.
5. Original data were provided.
6. These studies were published as full-text articles in English.

The exclusion criteria for the studies were as follows:

1. The study population consisted of older adults (aged ≥ 50 years) with a confirmed diagnosis of dementia, including but not limited to Alzheimer’s disease, vascular dementia, or mixed dementia. The diagnosis must have been made using established clinical criteria (e.g., DSM-IV, DSM-5, NINCDS-ADRDA, NIA-AA) or standardized assessment tools.
2. The intervention did not involve any form of physical activity.
3. No outcomes related to executive function were reported.
4. Study types included ineligible categories such as qualitative research, reviews, theses, and conference proceedings.
5. Non-interventional study designs, such as cross-sectional studies, case–control studies, and cohort studies, were excluded.
6. Articles not published in English full-text, studies with unavailable full texts, or incomplete data.
7. Confounding factors were excluded; patients with the following comorbidities might interfere with the assessment of executive function, such as severe somatic diseases (e.g., end-stage heart failure, advanced cancer), acute episodes of psychiatric disorders (e.g., major depressive episode, uncontrolled schizophrenia), and other neurological diseases (e.g., multiple sclerosis, post-stroke executive function impairments not analyzed separately). Furthermore, studies that exclusively enrolled populations with Mild Cognitive Impairment (MCI).
8. Published in a language other than English.

### 2.5 Data extraction

The data extraction process was independently carried out by two researchers (YZ and XLZ). Any discrepancies encountered during this process were resolved through group discussion. The following information was extracted from each study:

1. Initial extraction: Data information was independently extracted by two researchers to ensure objective collection of information.
2. Discrepancy resolution: Any discrepancies in the extracted data were resolved through group discussions until consensus was achieved.
3. Information categorization: The following four categories of data were systematically extracted from each study:

*Basic study information*: First author, publication year, and country/region where the study was conducted;

*Participant characteristics*: Age, total sample size, and group allocation;

*Intervention details*: Type of intervention, duration of intervention, weekly frequency, and total number of sessions; and

*Outcome measures*: Primary or secondary outcomes directly related to executive function in older adults with dementia and their corresponding measurement tools.

1. Special data handling principles: For numerical information presented graphically but ambiguously described in text, Engauge Digitizer 12.1 software was used for digital extraction.

When a study reported multiple follow-up time points, preference was given to data assessed immediately after the intervention ended. In the absence of standard deviation (SD), SD values were estimated using the recommended formula from the Cochrane Handbook, utilizing the 95% confidence interval of the group means. To ensure that effect sizes from different outcome measures were conceptually aligned before pooling, we harmonized the direction of effects. A positive standardized mean difference (SMD) was defined to consistently represent an improvement in executive function. For outcome measures where a decrease in score indicates improvement (e.g., Trail Making Test Part B [TMT-B] completion time, Stroop test interference time), the mean difference was multiplied by-1. For measures where an increase in score indicates improvement (e.g., Digit Span), the original values were retained.

### 2.6 Quality assessment

We utilized the Cochrane Risk of Bias Assessment Tool (RoB2) to evaluate the quality of the studies based on five criteria: (1) the randomization process; (2) deviations from the intended interventions; (3) missing outcome data; (4) measurement of outcomes; and (5) selection of reported results. Based on these criteria, we determined the overall risk of bias for each study, categorizing them as having low risk, high risk, or some concerns.

### 2.7 Statistical analysis

For continuous outcomes, we calculated the SMD and its corresponding 95% confidence intervals (CIs). To assess statistical heterogeneity, we employed the *p*-value from the Chi-square test and evaluated the I^2^ statistic, where an I^2^ value exceeding 50% typically indicates moderate heterogeneity, and values above 75% suggest high heterogeneity. Considering the diversity of scales used in the analysis, we applied a random-effects model to estimate overall differences, ensuring consistency and enhancing comparability. To address scale heterogeneity across studies (e.g., divergent measurement tools and intervention protocols), we implemented a random-effects model with inverse variance weighting, explicitly accounting for between-study variability through τ^2^ estimation. This conservative approach preserved methodological parsimony while enhancing comparative consistency. In accordance with PRISMA-NMA specifications, a frequentist framework was prioritized over Bayesian alternatives to optimize interpretability and avoid computational complexities associated with Markov chain Monte Carlo convergence. The analytical workflow encompassed three core components: network configuration using Stata 15.1’s ‘network’ package generated evidence diagrams where node diameters scaled with study sample sizes and connecting line thickness reflected trial counts per comparison; effect size synthesis via maximum likelihood estimation in multivariate meta-regression for integrating direct–indirect evidence; and consistency validation through node-splitting tests quantifying disagreement between direct and indirect comparisons (with *p* > 0.05 indicating statistical consistency).

Network meta-analysis was performed using a frequentist approach. To prepare the data, we employed the network package, which enabled us to generate evidence network plots. In these plots, each node represents a specific intervention, with the size of the node corresponding to the sample size of the related studies. Direct comparisons between interventions are represented by lines connecting the nodes, where the thickness of the lines reflects the number of studies included in each comparison; thicker lines indicate a larger number of studies. To assess the effectiveness of different interventions, we calculated the SUCRA and presented the results in a probability ranking table. SUCRA values, expressed as percentages, reflect the effectiveness of interventions, with higher percentages indicating more effective treatments. To evaluate potential publication bias, we constructed funnel plots and adjusted for the potential impact of publication bias on the results.

## 3 Results

### 3.1 Trial selection

To ensure the reliability of the literature search and screening process, two researchers (YZ and SL) independently reviewed the titles, abstracts, and full texts following the literature search. Cohen’s kappa coefficient was calculated to assess the inter-rater reliability for both stages of the screening: the title and abstract screening phase and the full-text screening phase, as well as the full-text screening phase. The consistency between the reviewers was categorized into three levels: moderate (0.40–0.59), good (0.60–0.74), and excellent (>0.75).

**Figure 1:**
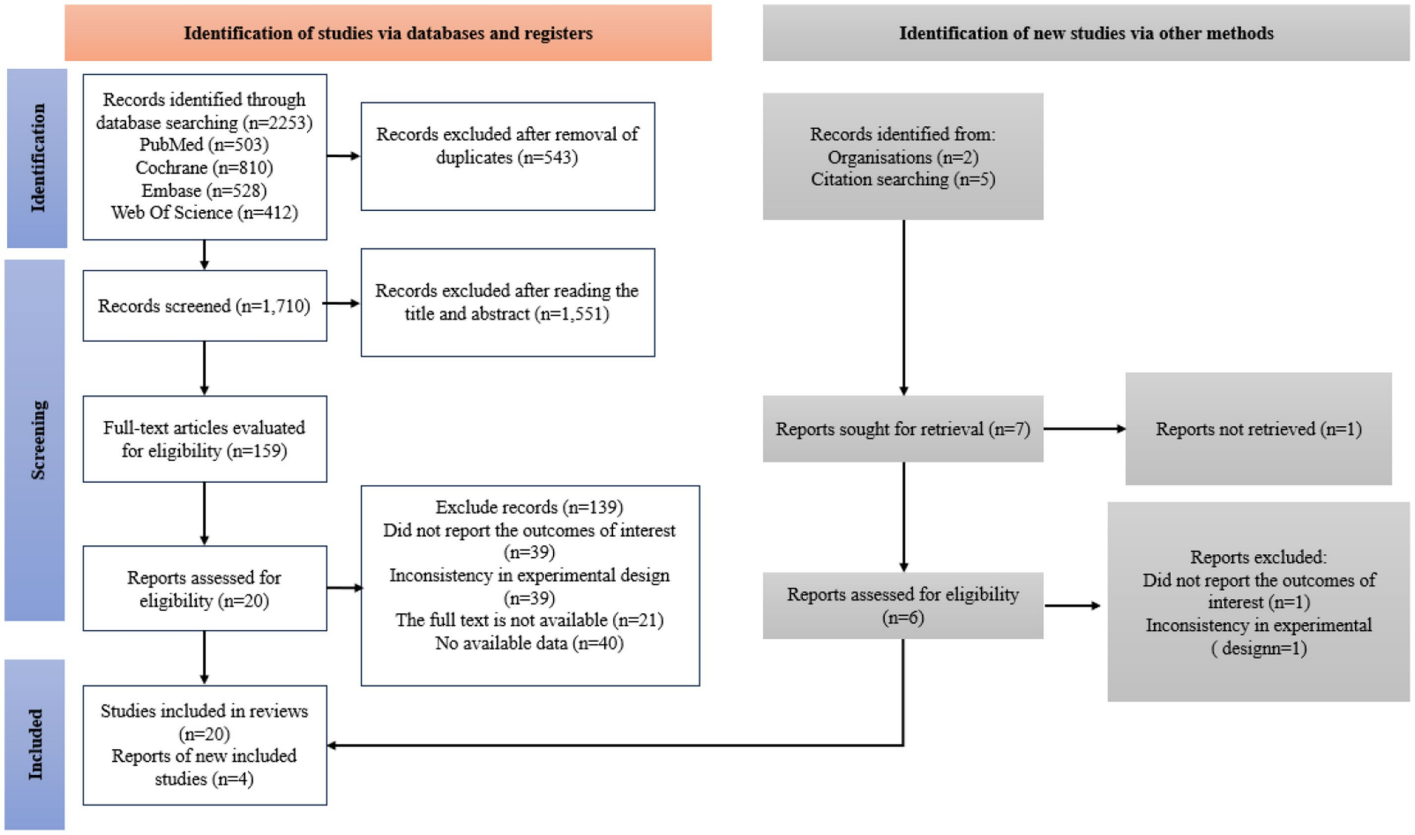
A summary of the evidence searches and selection process.

In the initial search, a thorough search was performed across four electronic databases covering the period from January 1, 2000, to May 4, 2025, resulting in the identification of 2,253 articles. After removing duplicates (*n* = 543), 1,710 articles remained for further evaluation. Through title and abstract screening, 1,551 articles were excluded, leaving 159 articles for full-text review. At this point, the inter-rater reliability between the two evaluators was deemed “good” (Cohen’s kappa = 0.73). Following the full-text review, 139 articles were further excluded: 39 did not report results, 39 had inconsistent experimental designs, 21 were unavailable in full text, and 40 lacked usable data. Consequently, 24 studies were included in the preliminary search (Figure 1). At this phase, the inter-rater reliability between the two evaluators was classified as “excellent” (Cohen’s kappa = 0.84).

### 3.2 Trial characteristics

The included studies encompassed participants with various types of dementia, primarily Alzheimer’s disease. The diagnosis and severity assessment were based on recognized criteria and tools, such as the Mini-Mental State Examination (MMSE), Clinical Dementia Rating (CDR), and others, as detailed in Table 1. Summarizes the characteristics of the studies included in the analysis. All studies were published between 2001 and 2025. The Netherlands had the largest number of publications, contributing five articles. The sample size of the intervention groups ranged from 14 to 116 participants, with a total of 1,202 individuals diagnosed with dementia. In the control groups, sample sizes ranged from 10 to 118 participants, comprising a total of 953 individuals with dementia. The average age of participants in both the experimental and control groups exceeded 70 years.

**Table 1:** Summary table of included reviews.

NO.	Study	Country	N (IG; CG)	Age (IG; CG)	Intervention (IG)			Intervention (CG)			Population	Outcomes
					Intervention content	Intervention time, frequency, period	Type	Intervention content	Intervention time, frequency, period	Type		
1	L. M. J. Sanders et al., 2020 (51)	Netherlands	39; 30	81.7 (7.16); 82.1 (7.51)	Walking sessions and Lower limb strength exercises	30 min each time, 3 times a week, 24 weeks.	Resistance exercise	Flexibility exercises and recreational activities	30 min each time, 3 times a week, 24 weeks.	Stretching exercise	Older persons with mild–moderate dementia	MMSE
2	Marinda Henskensa et al., 2018 (46)	Netherlands	22; 22	85.14 ± 4.64; 84.73 ± 4.55	Alternating strength exercise (chest press)and aerobic exercise (outdoor walking)	30–45 min each time, 3 times a week, 6 month.	Multi-mode motion	Usual care	30 min each time, 3 times a week, 6 month	Usual care	Residents with moderate to severe dementia	MMSE
3	Cynthia Arcoverde et al., 2014 (36)	Brazil	20; 10	78.5 (64–81.2); 79 (74.7–82.2)	Treadmill walking	30 min each time, 2 times a week, 4 month.	Aerobic exercise	Usual care	4 month	Usual care	Mild Alzheimer’s disease patients	CDT
4	Nicole Dawson et al., 2019 (53)	USA	13; 10	73.8 (8.5); 74.0 (10.4)	Strength and balance exercises.	2 times a week, 12 weeks.	Resistance exercise	Usual care	12 weeks	Usual care	Individuals with mild–moderate dementia	TMT-B
5	Lievyn Enette et al., 2020 (25)	France	14; 21	74 (68–83); 79 (75–84)	Constant aerobic cycling	30 min each time, 2 times a week, 9 weeks.	Aerobic exercise	Health communication course	30 min each time, 1 times a week, 9 weeks.	Psychotherapy	Alzheimer’s disease patients	MMSE
5	Lievyn Enette et al., 2020 (37)	France	17; 21	79 (75–82); 79 (75–84)	Intermittent aerobic cycling	30 min each time, 2 times a week, 9 weeks.	Aerobic exercise	Health communication course	30 min each time, 1 times a week, 9 weeks.	Psychotherapy	mild–moderate Alzheimer’s disease patients	MMSE
6	Cristina Fonte et al., 2019 (27)	Italy	20; 21	79 ± 9;80 ± 7	Physical Treatment: moderate intensity endurance (cycling, walking) and resistance (chest-press) training	90 min each time, 3 times a week, 6 month.	Multi-mode motion	Usual care	24 weeks	Usual care	Alzheimer’s disease patients	ADAS-Cog
7	Kristine Hoffmann et al., 2016 (38)	Denmark	107; 93	69.8 ± 7.4; 71.3 ± 7.3	moderate-to high intensity aerobic exercise (on an ergometer bicycle, cross trainer, and treadmill)	60 min each time, 3 times a week, 16 weeks.	Aerobic exercise	Usual care	16 weeks	Usual care	patients with mild–moderate Alzheimer’s disease	Stroop
8	Vjera A. Holthoff et al., 2015 (54)	Germany	15; 15	72.4 ± 4.3; 70.67 ± 5.41	Resistance exercise	60 min each time, 3 times a week, 12 weeks.	Resistance exercise	Usual care	12 weeks	Usual care	Patients with Alzheimer’s disease	MMSE
9	Nayan Huang et al., 2019 (26)	China	40; 40	81.9 ± 6.0; 81.9 ± 6.1	Tai Chi	3 times a week, 10 month.	Mind–body exercise	Usual care	10 month	Usual care	Older persons with mild dementia	MMSE
10	Esther G. A. Karssemeijer et al., 2019 (39)	Netherlands	38; 39	79.0 (6.9); 79.8 (6.5)	The exergame training consisted of a combined cognitive–aerobic bicycle training	30–50 min each time, 3 times a week, 12 weeks.	Sensory-motor training	Relaxation and flexibility exercises	30 min each time, 3 times a week, 12 weeks.	Stretching exercise	People with dementia	MMSE
10	Esther G. A. Karssemeijer et al., 2019 (39)	Netherlands	38; 39	80.9 (6.1); 79.8 (6.5)	Aerobic bikes	30–50 min each time, 3 times a week, 12 weeks.	Aerobic exercise	Relaxation and flexibility exercises	30 min each time, 3 times a week, 12 weeks.	Stretching exercise	People with dementia	MMSE
11	Jill K. Morris et al., 2017 (40)	Australia	39; 38	74.4 (6.7); 71.4 (8.4)	Moderate intensity aerobic exercise	150 min each time, 3–5 times a week,26 weeks.	Aerobic exercise	Core strengthening, resistance bands, modified tai chi, modified yoga	150 min each time, 3–5 times a week,26 weeks.	Mind–body exercise	Mild–moderate Alzheimer’s disease patients	CSD
12	Hannareeta Ohman et al., 2016 (41)	Finland	70; 70	77.7 ± 5.4; 78.1 ± 5.3	Home-Based Exercise: Aerobic, training, Strength and endurance, Balance training	60 min each time, 2 times a week, 12 month.	Multi-mode motion	Usual care	12 month	Usual care	Alzheimer’s disease patients	CDT
12	Hannareeta Ohman et al., 2016 (41)	Finland	70; 70	78.3 ± 5.1; 78.1 ± 5.3	Group Exercise: Aerobic, training, Strength and endurance, Balance training	60 min each time, 2 times a week, 12 month.	Aerobic exercise	Usual care	12 month	Usual care	Alzheimer’s disease patients	CDT
13	Anna-Eva Prick et al., 2017 (47)	Netherlands	57; 54	76 (7.61); 78 (7.17)	Exercise training four types of exercises (flexibility, strengthening, balance and endurance)	30 min each time, 3 times a week, 3 month.	Multi-mode motion	Usual care	3 month	Usual care	People With Dementia	CDT
14	Felipe de Oliveira Silva et al., 2017 (41)	Brazil	13; 14	81.22 ± 8.88; 77.54 ± 8.05	Multimodal physical exercises (aerobic, strength, balance and flexibility)	60 min each time, 2 times a week, 12 weeks.	Aerobic exercise	Usual care	12 weeks	Usual care	Elderly individuals with Alzheimer’s disease	CDR
15	Annika Toots et al., 2017 (48)	Sweden	107; 142	84.4 (6.2); 85.9 (7.8)	High Intensity Functional Exercise (HIFE)	45 min each time, 5 times every 2 weeks, 4 months.	Multi-mode motion	Usual care	4 months	Usual care	Older People With moderate to severe Dementia	MMSE
16	Lidia Ya’guez et al., 2010 (52)	UK	15; 12	70.5; 75.5	Non-aerobic movement (Brain Gym1 Program)	120 min each time, 6 weeks.	Stretching exercise	Usual care	6 weeks	Usual care	Alzheimer’s type dementia	ICD
17	Fang Yu et al., 2021 (43)	USA	64; 32	77.4 ± 6.6; 77.5 ± 7.1	Cycling exercise	20–50 min each time, 3 times a week, 6 month.	Aerobic exercise	Stretching	6 month	Stretching exercise	Older adults with Alzheimer’s disease dementia	ADAS-Cog
18	LCW Lam et al., 2021 (44)	China	94; 94	80.3 ± 6.2; 80.8 ± 6.3	Physical exercise	45 min each time, 2 times a week, 6 month.	Aerobic exercise	Health education	45 min each time, 2 times a week, 6 month.	Psychotherapy	Elders with mild clinical Alzheimer disease	ADAS-Cog
19	Sandra Trautwein et al., 2021 (49)	Germany	201; 118	85 ± 7; 86 ± 5	Multi-modal exercise program and Motor and cognitive tasks (contained tasks in standing position and specific walking exercises.)	2 times a week, 16 weeks.	Multi-mode motion	Usual Care	2 times a week, 16 weeks.	Usual care	Elders with mild clinical Alzheimer disease	MMSE
20	Pengfei Wang et al., 2014 (56)	China	62; 61	66.39 ± 4.24; 67.82 ± 4.81	Cognitive training and lifestyle guidance (Baguanjin)	90 min each time, 1 time a week, 7 weeks.	Mind–body exercise	Usual Care	7 weeks	Usual Care	Individuals with mild dementia	MMSE
21	Shanshan Wu et al., 2023 (45)	Korea	13; 11	78.8; 81.2	EXG engaged in a running-based exergame	30–50 min each time, 3 times a week, 12 weeks.	Sensory-motor training	Cycling exercise	30–50 min each time, 3 times a week, 12 weeks.	Aerobic exercise	Older persons with dementia	CERAD-K
22	Aoyu Li et al., 2025 (57)	China	116; 116	73.03; 72.7	motion-sensing exercises (such as waving, jumping, arm swinging,)	60 min each time, 2 times a week, 12 weeks.	Sensory-motor training	Usual care	60 min each time, 2 times a week, 12 weeks.	Usual care	Individuals with mild-moderate dementia	MMSE
23	Låtta Hasselgren et al., 2024 (55)	Sweden	31; 29	78.4 ± 6.0; 79.0 ± 7.1	Group physical exercise (lower-limb strength exercises, Balance exercises)	45 min each time, 2 times a week, 16 weeks.	Resistance exercise	Usual care	20 weeks	Usual care	Older persons with mild dementia	GDS
24	Shari David et al., 2025 (50)	Germany	22; 19	72.1 ± 5.8; 68 ± 8.2	Exercise interventions, including aerobic exercise, strength training, and coordination training	60 min each time, 1 time a week, 26 weeks.	Multi-mode motion	Psychoeducational programs	1 time a month,26 weeks	Psychotherapy	Mild Alzheimer’s disease patients	BDI

IG, intervention group; CG, control group; N, Number; NA, not available; MMSE, Mini Mental State Examination; CDT, Clock Drawing Test; TMT-B, Trail Making Test—Part B; ADAS-Cog, Cognitive section of the Alzheimer’s Disease Assessment Scale; ST, Stroop task; CSD, Cornell Scale for Depression; CDR, Clinical Dementia Rating scale; ICD, International Statistical Classification of Diseases and Related Health Problems; CERAD-K, Consortium to Establish a Registry for Alzheimer’s Disease-Korean; GDS, Geriatric Depression Scale; BDI, Beck Depression Inventory.

To examine whether various forms of physical activity exert different effects on executive function in older adults with dementia, we categorized the activities into six groups based on shared characteristics and findings from prior studies (34, 35). This classification was developed through discussions within the research team and consultations with experts. The six categories include aerobic exercise (10 studies) (25, 36–44), which primarily involves continuous, rhythmic physical activities aimed at improving cardiovascular endurance (e.g., treadmill walking, cycling, brisk walking); multi-mode motion (7 studies) (24, 27, 40, 45–48), referring to interventions that explicitly combined two or more distinct categories of exercise (e.g., aerobic + resistance, resistance + balance) within a single, integrated program with comparable dosage for each component; stretching exercise (4 studies) (38, 42, 49, 50), which primarily involves low-intensity activities aimed at improving flexibility and range of motion, often serving as an active control in many studies; resistance exercise (4 studies) (49, 51–53), aimed at enhancing muscular strength and endurance through exercises against resistance; mind–body exercise (3 studies) (26, 39, 54), which combines physical movement, mental focus, and controlled breathing to promote harmony between body and mind; and sensory-motor training (3 studies) (38, 44, 55), primarily utilizing exergames or technology-based platforms that simultaneously engage cognitive and motor functions through interactive tasks.

Non-physical activity interventions include psychological interventions and usual care. Commonly used measurement tools include the Mini-Mental State Examination, Clock Drawing Test, Cornell Scale for Depression, and the Alzheimer’s Disease Assessment Scale – Cognitive Subscale, among others.

### 3.3 Risk of bias

**Figure 2:**
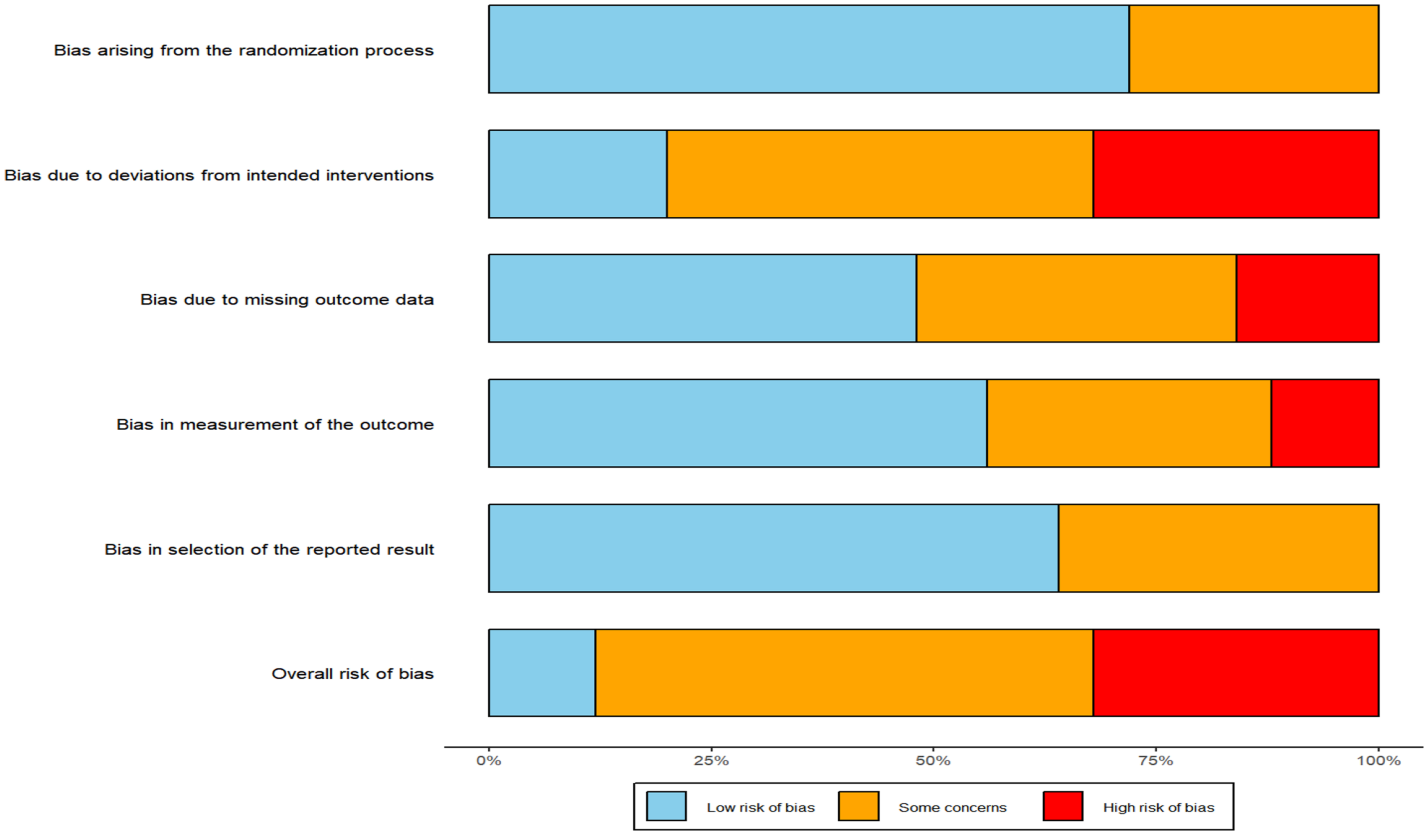
Risk of bias of included studies.

Among the 24 studies, 10 were determined to have a low risk of bias in terms of randomization, while 14 did not provide sufficient details on the randomization process. In terms of deviations from the intended interventions and missing outcome data, 6 studies were rated as having a low risk of bias, 9 studies had a high risk, and 9 studies showed moderate issues. Regarding bias in outcome measurement, 11 studies were classified as having a low risk, 2 studies as having a high risk, and 11 studies presented moderate concerns. As for selective reporting bias, 20 studies were rated as having a low risk, 4 studies showed some issues, and none were classified with a high risk. Evaluating these five criteria collectively, the overall risk of bias across the 24 studies was distributed as follows: 11 studies showed moderate concerns, 9 studies were assessed with a high overall risk of bias, and 4 studies were classified as having a low risk. The detailed results of the bias assessment are presented in Figure 2 and Appendix C, which provide a comprehensive breakdown of each study’s ratings and classifications across the various bias risk criteria.

### 3.4 Network meta-analysis

**Figure 3:**
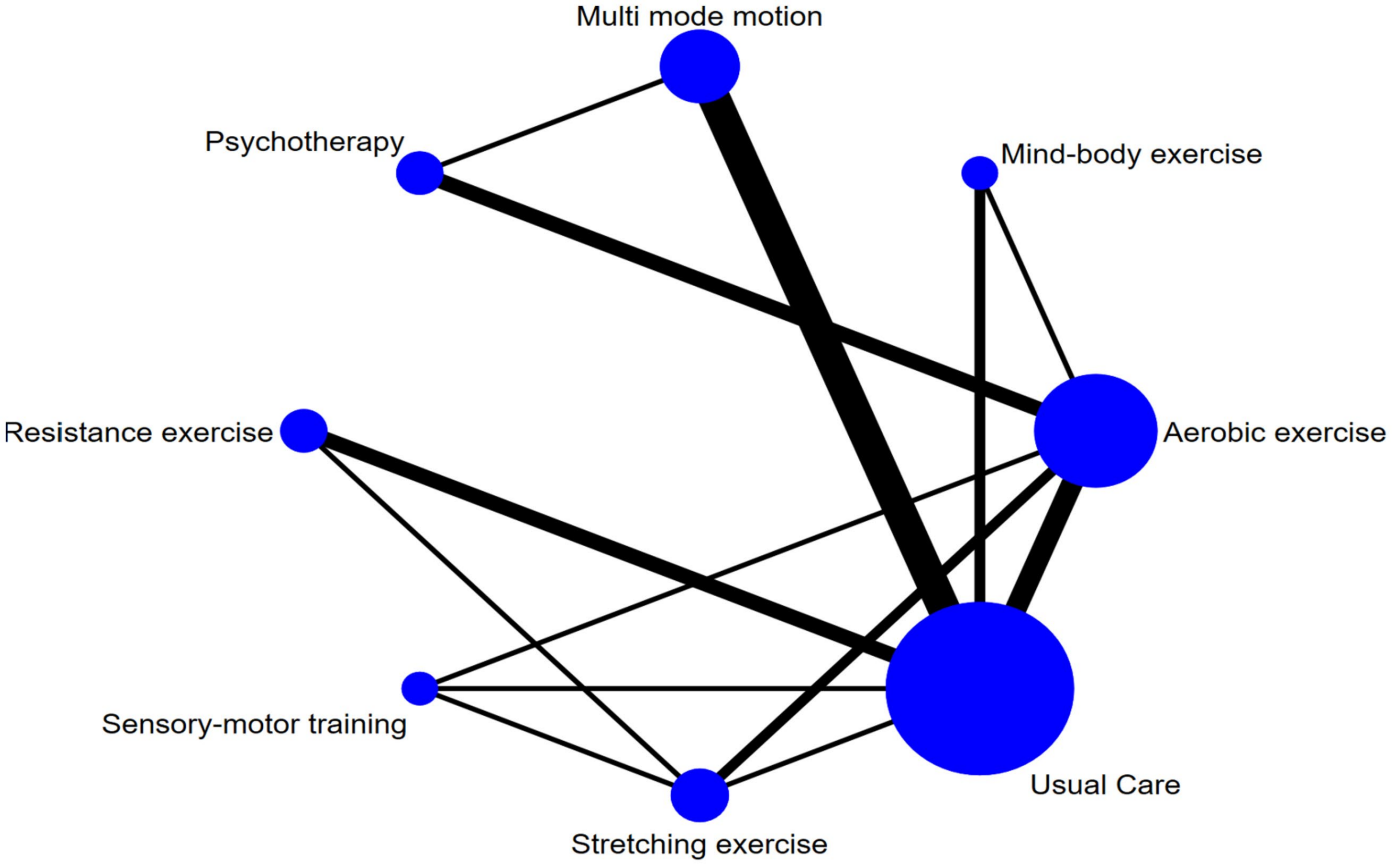
Network diagram.

Figure 3 presents the network meta-analysis diagram. The three interventions with the largest sample sizes in the experimental group were aerobic exercise, multi-mode motion, and resistance exercise. In contrast, the intervention with the largest sample size in the control group was usual care. The most frequently studied comparisons included traditional aerobic exercise versus usual care and multi-mode motion versus usual care.

**Figure 4:**
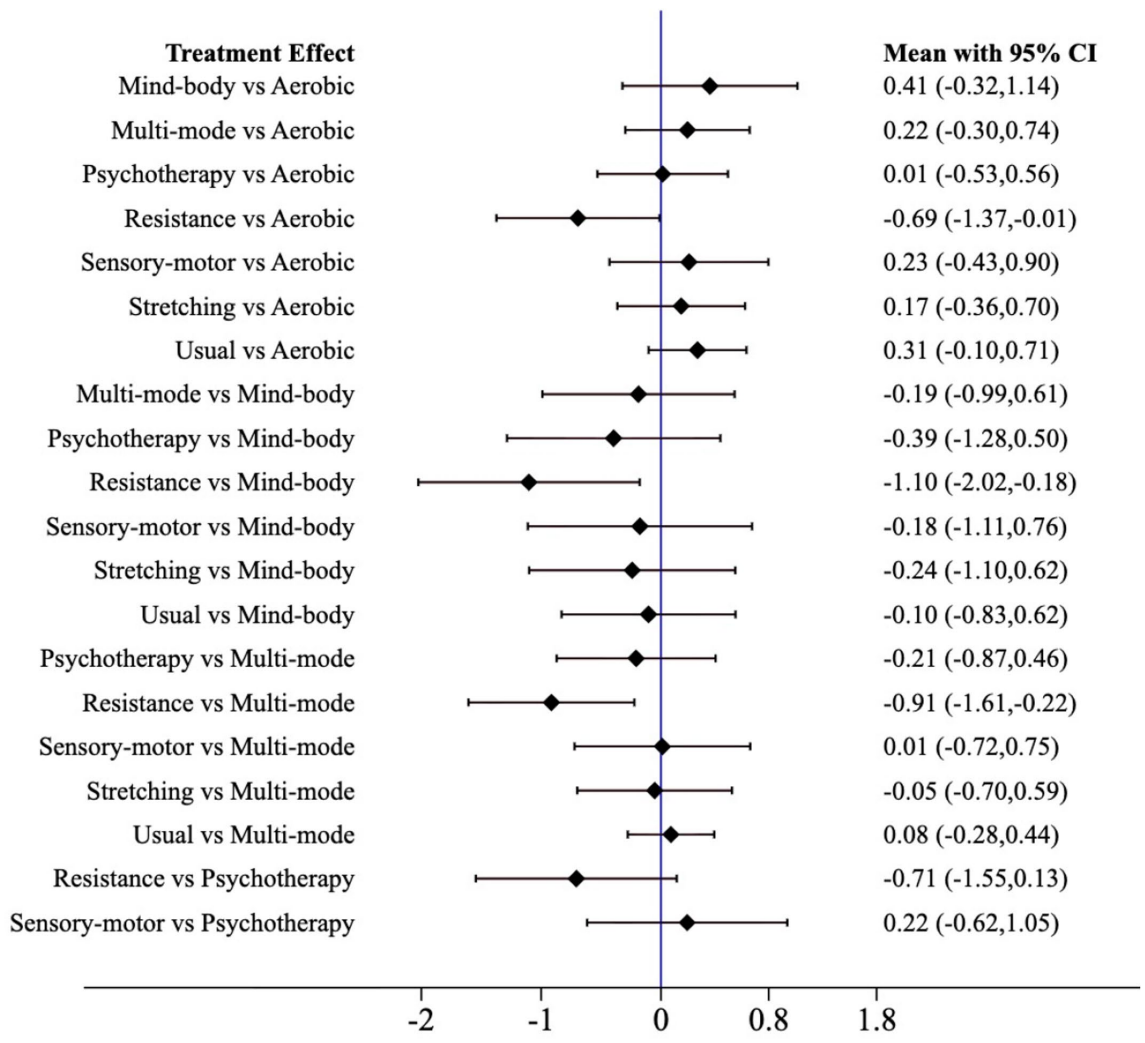
Forest plot.

The forest plot compares the standardized mean differences (SMD) with 95% confidence intervals (95% CIs) of physical activity interventions for executive function in older adults with dementia, and presents direct and indirect analyses (Figure 4). Aerobic exercise and multi-mode motion are more effective than control conditions; a higher SMD value indicates a better therapeutic effect.

Resistance exercise demonstrated superior improvement outcomes: compared to mind–body exercise [SMD = 0.41 (95% CI: −0.87, 1.68)], aerobic exercise [SMD = 0.63 (95% CI: −0.50, 1.77)], sensory-motor training [SMD = 0.68 (95% CI: −0.72, 2.08)], multi-mode motion [SMD = 0.71 (95% CI: −0.36, 1.78)], stretching exercise [SMD = 1.38 (95% CI: −0.11, 2.88)], and psychotherapy [SMD = 1.48 (95% CI: −0.32, 3.29)], resistance exercise showed an improvement advantage, with a significant improvement compared to usual care [SMD = 1.16 (95% CI: 0.28, 2.03)]. Mind–body exercise, when compared to aerobic exercise [SMD = 0.23 (95% CI: −0.74, 1.20)], sensory-motor training [SMD = 0.27 (95% CI: −1.15, 1.70)], multi-mode motion [SMD = 0.31 (95% CI: −0.81, 1.42)], stretching exercise [SMD = 0.98 (95% CI: −0.57, 2.53)], and psychotherapy [SMD = 1.08 (95% CI: −0.65, 2.80)], also showed an improvement advantage. Aerobic exercise, compared to sensory-motor training [SMD = 0.05 (95% CI: −1.20, 1.29)], multi-mode motion [SMD = 0.08 (95% CI: −0.90, 1.05)], stretching exercise [SMD = 0.75 (95% CI: −0.58, 2.08)], psychotherapy [SMD = 0.85 (95% CI: −0.60, 2.30)], and usual care [SMD = 0.52 (95% CI: −0.24, 1.28)], showed an improvement advantage. Multi-mode motion, compared to stretching exercise [SMD = 0.67 (95% CI: −0.80, 2.15)], psychotherapy [SMD = 0.77 (95% CI: −0.91, 2.46)], and usual care [SMD = 0.45 (95% CI: −0.17, 1.07)], demonstrated an improvement advantage. Sensory-motor training, compared to stretching exercise [SMD = 0.71 (95% CI: −0.68, 2.09)], psychotherapy [SMD = 0.81 (95% CI: −1.08, 2.69)], and usual care [SMD = 0.48 (95% CI: −0.67, 1.63)], also exhibited an improvement advantage. The specific results are presented in Table 2.

**Table 2:** League table on interventions.

Resistance exercise	Mind–body exercise	Aerobic exercise	Sensory-motor training	Multi-mode motion	Usual care	Stretching exercise	Psychotherapy
Resistance exercise	−0.41 (−1.68,0.87)	−0.63 (−1.77,0.50)	−0.68 (−2.08,0.72)	−0.71 (−1.78,0.36)	−1.16 (−2.03,−0.28)	−1.38 (−2.88,0.11)	−1.48 (−3.29,0.32)
0.41 (−0.87,1.68)	Mind–body exercise	−0.23 (−1.20,0.74)	−0.27 (−1.70,1.15)	−0.31 (−1.42,0.81)	−0.75 (−1.70,0.19)	−0.98 (−2.53,0.57)	−1.08 (−2.80,0.65)
0.63 (−0.50,1.77)	0.23 (−0.74,1.20)	Aerobic exercise	−0.05 (−1.29,1.20)	−0.08 (−1.05,0.90)	−0.52 (−1.28,0.24)	−0.75 (−2.08,0.58)	−0.85 (−2.30,0.60)
0.68 (−0.72,2.08)	0.27 (−1.15,1.70)	0.05 (−1.20,1.29)	Sensory-motor training	−0.03 (−1.32,1.26)	−0.48 (−1.63,0.67)	−0.71 (−2.09,0.68)	−0.81 (−2.69,1.08)
0.71 (−0.36,1.78)	0.31 (−0.81,1.42)	0.08 (−0.90,1.05)	0.03 (−1.26,1.32)	Multi-mode motion	−0.45 (−1.07,0.17)	−0.67 (−2.15,0.80)	−0.77 (−2.46,0.91)
1.16 (0.28,2.03)	0.75 (−0.19,1.70)	0.52 (−0.24,1.28)	0.48 (−0.67,1.63)	0.45 (−0.17,1.07)	Usual care	−0.23 (−1.58,1.12)	−0.33 (−1.92,1.27)
1.38 (−0.11,2.88)	0.98 (−0.57,2.53)	0.75 (−0.58,2.08)	0.71 (−0.68,2.09)	0.67 (−0.80,2.15)	0.23 (−1.12,1.58)	Stretching exercise	−0.10 (−2.05,1.85)
1.48 (−0.32,3.29)	1.08 (−0.65,2.80)	0.85 (−0.60,2.30)	0.81 (−1.08,2.69)	0.77 (−0.91,2.46)	0.33 (−1.27,1.92)	0.10 (−1.85,2.05)	Psychotherapy

**Figure 5:**
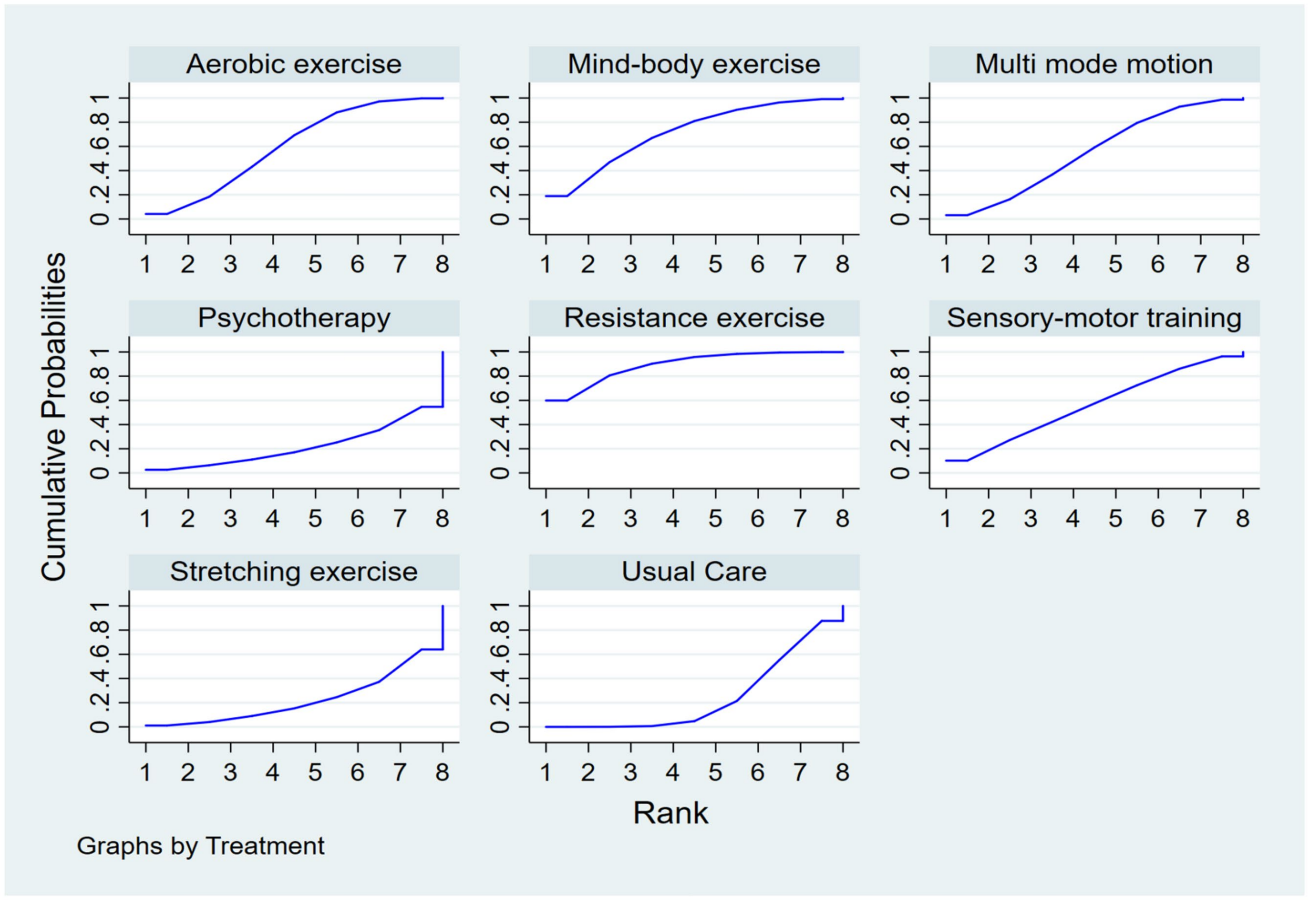
SUCRA plot.

Regarding the probability of different interventions’ effects on executive function in older adults with dementia, based on the SUCRA index, the first tier (best effects) includes resistance exercise, with a SUCRA of 89.2%, PrBest of 59.9%, and an average ranking of 1.8, making it the most likely effective intervention. Mind–body exercise follows with a SUCRA of 71.4%, PrBest of 18.9%, and an average ranking of 3.0, showing significant effects. The second tier (moderate effects) includes aerobic exercise with a SUCRA of 60.0%, PrBest of 4.2%, and an average ranking of 3.8, serving as the benchmark for comparison. Sensory-motor training and multi-mode motion have similar effects, with SUCRA values of 56.0 and 55.2%, PrBest values of 10.2 and 3.1%, and an average ranking of 4.1, ranking them jointly in fourth place. Specific results are shown in Figure 5.

### 3.5 Publication bias

**Figure 6:**
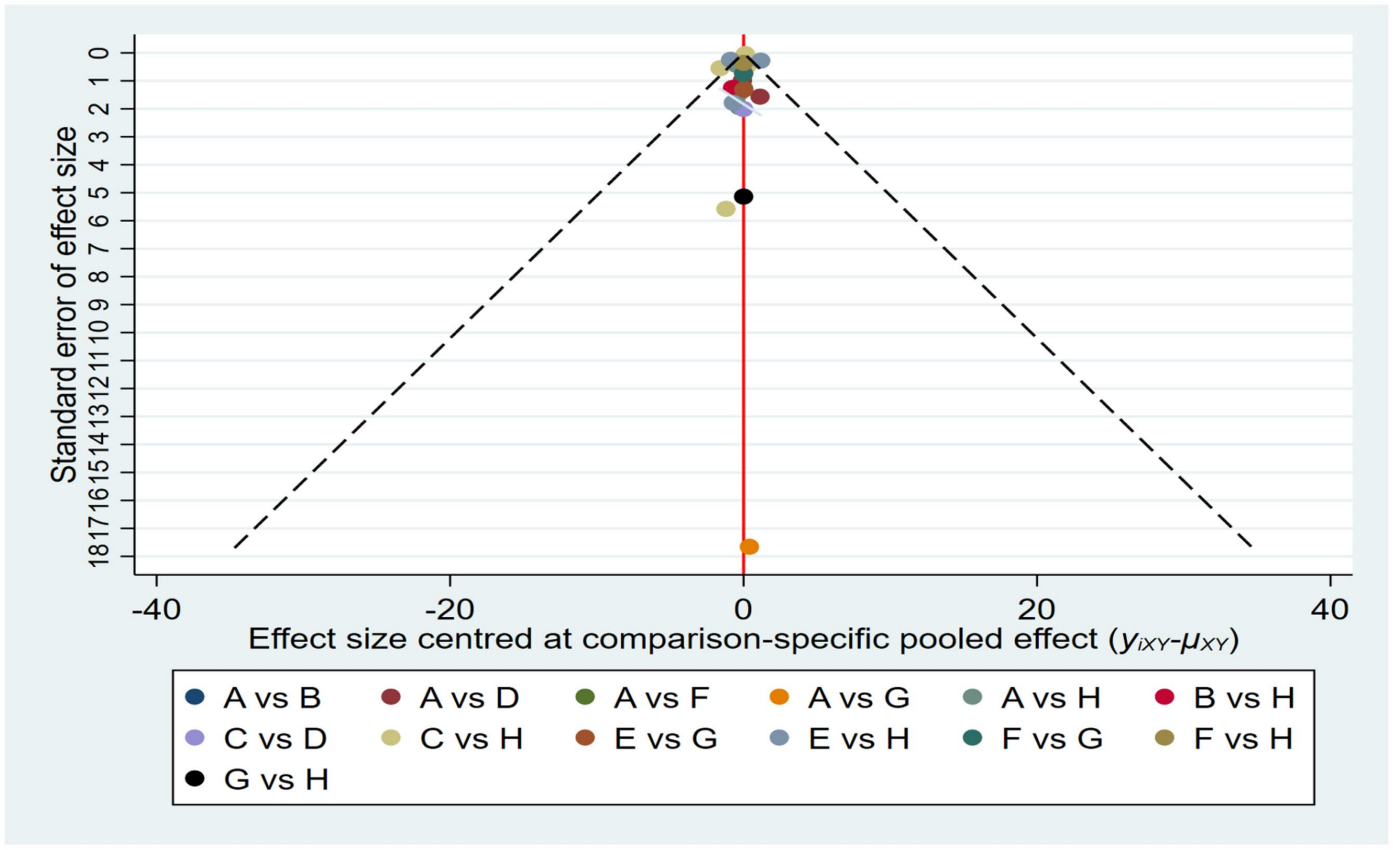
Funnel plot on publication bias.

As shown in Figure 6, we initially assessed publication bias using a funnel plot. The distribution of studies in the funnel plot appears approximately symmetrical, and visual inspection did not reveal any obvious signs of publication bias. This suggests that while some degree of publication bias may be present in the original data, its impact is not significant. Overall, the estimated effect sizes remain statistically meaningful, indicating the robustness of the study results.

## 4 Discussion

Executive function, as a core component of higher cognitive abilities, is particularly impaired in older adults with dementia, significantly affecting their daily living skills and disease progression (56). Physical activity, due to its high safety profile and low side effects, has become a focal point in research aimed at delaying cognitive decline (57, 58). However, the effectiveness of different physical activity modes in improving executive function remains controversial (59, 60), highlighting the need for an evidence-based comprehensive evaluation. This network meta-analysis provides a thorough assessment of the effects of various physical activity interventions on executive function in dementia. Through a comprehensive analysis of 24 RCTs, the findings indicate that resistance exercise, mind–body exercise, and aerobic exercise are the most effective non-pharmacological interventions for improving executive function in this population.

Methodological Considerations and Clinical Heterogeneity. Some methodological aspects of the included studies warrant discussion. Firstly, the diagnosis of dementia in all included trials was based on established clinical criteria, such as the Diagnostic and Statistical Manual of Mental Disorders or the National Institute of Neurological and Communicative Disorders and Stroke–Alzheimer’s Disease and Related Disorders Association criteria, as performed by physicians or specialized clinicians (33). While cognitive screening tools like the MMSE were frequently reported, they served primarily for assessing baseline cognitive severity or monitoring change, not as standalone diagnostic tools. The severity of dementia was commonly assessed using standardized measures, including the CDR scale and the MMSE itself, which allowed for a rough stratification (e.g., mild vs. moderate) in several studies (42, 49). However, it must be acknowledged that the exercise protocols (including type, frequency, intensity, and duration) were largely standardized within each study and were not typically individually tailored based on dementia severity levels (26). This lack of stratification limits our ability to perform a definitive subgroup analysis on whether efficacy differs between mild versus severe dementia (32). Anecdotally, interventions like aerobic exercise appeared to show more promise in individuals with mild MCI or early-stage dementia (25, 43), whereas resistance exercise demonstrated benefits across a broader spectrum (49, 51–53). This suggests that the optimal type of physical activity may be dependent on the patient’s baseline cognitive and physical function (30). Future studies should explicitly stratify participants by severity using robust tools like the CDR and design adaptive interventions to determine the most effective exercise prescription for each stage of dementia (41). Notably, resistance exercise is considered the most likely intervention to yield the best results (SUCRA = 89.2%), followed by mind–body exercise (SUCRA = 71.4%), with aerobic exercise ranking third (SUCRA = 60.0%) (Table 3). It is important to note that the SUCRA value represents a probabilistic ranking of the interventions rather than a direct measure of clinical effect size. A higher SUCRA indicates a greater probability that an intervention is among the best, but this interpretation should be considered alongside the estimated effect sizes presented in the league table (Table 2).

**Table 3:** Ranking of SUCRA probabilities.

Intervention name	SUCRA (%)	PrBest (%)	Mean rank
Resistance exercise	89.2	59.9	1.8
Mind–body exercise	71.4	18.9	3
Aerobic exercise	60	4.2	3.8
Sensory-motor training	56	10.2	4.1
Multi-mode motion	55.2	3.1	4.1
Usual Care	24.3	0	6.3
Stretching exercise	22.2	1.1	6.4
Psychotherapy	21.8	2.6	6.5

SUCRA, Surface Under the Cumulative Ranking curve. Higher SUCRA values indicate a higher probability of being among the most effective treatments. PrBest indicates the probability that the treatment is the best. Mean Rank reflects the average ranking position across simulations. Represents the ranking of interventions.

The mechanisms through which resistance exercise impacts executive function in dementia are primarily reflected in the improvement of neuroplasticity and the expression of brain-derived neurotrophic factor (BDNF), as well as in the promotion of neurogenesis and neuroprotection. This leads to enhanced brain structure and function, improved executive function, and better daily living abilities and quality of life (49, 51). Resistance exercise enhances skeletal muscle contraction, promotes neuroplasticity, and increases BDNF expression, which in turn supports neuronal growth and regeneration, thereby improving executive function and delaying neurodegenerative changes associated with dementia (52, 53). Moreover, resistance exercise can delay neurogenesis, exert neuroprotective effects, reduce the accumulation of *β*-amyloid plaques, lower inflammation, and safeguard brain health (61). By increasing cortical thickness in the frontal lobe and reducing white matter atrophy, resistance exercise further enhances executive and memory functions, while improving cerebral blood flow and strengthening cognitive abilities (62). Studies have shown that resistance exercise significantly impacts executive function in dementia, particularly by enhancing attention, inhibitory control, and multitasking abilities (49, 51–53). Additionally, resistance exercise contributes to the improvement of patients’ daily living abilities, reduces the risk of falls, and consequently enhances quality of life (63). Importantly, the positive effects of resistance exercise tend to persist over an extended period, with executive improvements maintained even after the intervention ends. Overall, resistance exercise, through various neurobiological mechanisms, serves as an effective non-pharmacological intervention for improving executive function in dementia.

Mind–body exercises, such as Tai Chi and Baduanjin, are also supported by significant neural foundations in improving emotional executive function (26, 39, 54). By modulating the nervous system and enhancing brain functional connectivity, mind–body exercises positively influence executive function. The practice of Tai Chi and Baduanjin strengthens the functional connectivity of the prefrontal cortex and medial prefrontal cortex, promoting the function of executive control networks, which is crucial for improving emotional regulation and cognitive function (64). Furthermore, these exercises increase the gray matter volume in the insula, medial temporal lobe, and caudate nucleus, regions that are closely associated with working memory and emotional regulation. Mind–body exercises help alleviate symptoms of anxiety and depression, regulate the autonomic nervous system, and enhance concentration and emotional states. At the same time, these practices enhance brain plasticity, improving the flexibility and adaptability of neural networks, thereby enhancing an individual’s ability to suppress negative emotions (65). Regarding executive function, Tai Chi and Baduanjin improve executive function by enhancing attention control, working memory, and cognitive flexibility (66). Research has shown that practicing Baduanjin significantly improves performance on logical memory and mental rotation tests, demonstrating its positive impact on cognitive control and information processing abilities (67). Overall, mind–body exercises provide a solid neural basis for improving emotional executive function by regulating the nervous system, enhancing brain functional connectivity, improving emotional regulation, and enhancing executive function.

Aerobic exercise exhibits certain limitations and varies in its applicable scenarios within dementia interventions (68). Studies have shown that aerobic exercise has a limited effect on improving executive function in individuals with moderate to severe dementia, and its effectiveness may be influenced by factors such as exercise adherence, duration, and individual differences (25, 36–44). In patients with early-stage dementia, aerobic exercise can delay cognitive decline and improve physical function and quality of life, particularly showing positive effects in reducing the risk of dementia (25, 41, 43). However, aerobic exercise demonstrates limited improvements in specific cognitive domains, such as language and visuospatial skills (69). Therefore, despite the limitations of aerobic exercise in dementia interventions, it holds substantial potential, especially in early-stage dementia, in improving physical function, quality of life, and in preventing dementia risk.

Multi-mode motion and sensory-motor training exhibit dual effects in terms of cognitive load. Multimodal feedback, by integrating stimuli such as visual and auditory cues, enhances motor perception, thereby improving task performance (24, 27, 40, 45–48). Studies have shown that multi-mode motion training can improve motor function, promote neuroplasticity, and increase cerebral blood flow, leading to enhanced executive function (70). However, when tasks are associated with high cognitive load, multimodal feedback may increase the cognitive load (71), thus impairing motor performance. Therefore, careful management of cognitive load is necessary when applying multimodal feedback (72).

Overall, resistance exercise, mind–body exercise, and aerobic exercise all demonstrate positive effects in improving executive function in older adults with dementia; however, their effectiveness and applicable contexts vary. Resistance exercise is particularly effective, showing more direct and significant improvements in both cognitive and executive functions. Mind–body exercise has unique advantages in enhancing emotional executive function and emotional regulation. Aerobic exercise demonstrates substantial potential, particularly in early-stage dementia, especially in terms of dementia risk prevention and improving quality of life. Multi-mode motion and sensory-motor training, however, should be used with caution to avoid excessive cognitive load that could impair task performance.

## 5 Strengths and limitations

This study offers several notable advantages. First, it is the pioneering network meta-analysis designed to investigate the effects of physical activity on executive function in individuals with dementia, providing crucial scientific evidence to inform the selection of suitable physical activity interventions for enhancing executive function in this group. Additionally, the synthesis of multiple studies substantially bolstered the reliability and precision of the findings. Moreover, the emphasis on RCTs, while intentionally excluding observational and cross-sectional studies, further enhanced the robustness of the conclusions. Nonetheless, certain limitations should be acknowledged. For example, individual variations among patients may lead to different responses to physical activity interventions, and factors such as the intensity and duration of physical activity could influence the overall effectiveness of these interventions.

Future studies could concentrate on several critical areas. Initially, developing personalized physical activity interventions that are customized to the specific needs of individuals with dementia may yield more effective results. For instance, when selecting appropriate interventions, factors such as the patient’s physical condition, medical history, and disease severity should be considered. Physical activity programs customized based on these individual variables could enhance their effectiveness in promoting the recovery of executive function. Additionally, further research is needed to explore the optimal parameters of physical activity, such as frequency, intensity, and duration, which are crucial for fine-tuning intervention strategies and maximizing executive function in dementia. Furthermore, although we established *a priori* rules for intervention categorization and verified their consistent application, some misclassification might remain possible due to the varying reporting details across included studies. However, our robustness check showed that the network structure was stable to the classification process.

## 6 Conclusion

This study confirms that resistance exercise (SUCRA = 89.2%, average rank = 1.8) and mind–body exercise (SUCRA = 71.4%, average rank = 3.0) are the most effective non-pharmacological interventions for improving executive function in dementia patients, with aerobic exercise (SUCRA = 60.0%) being the next most effective. In clinical practice, resistance exercise is recommended as the primary intervention, or, based on individual patient characteristics such as dementia severity and physical capability, mind–body or aerobic exercise can be chosen. Additionally, intervention parameters such as frequency and intensity should be standardized to enhance reproducibility. Future research should focus on the development of personalized exercise programs (incorporating genetic, phenotypic, and cognitive baselines), long-term efficacy observations, and studies on intervention mechanisms such as brain-derived neurotrophic factor expression and prefrontal cortex connectivity to further optimize strategies for improving executive function in dementia patients.

## Data Availability

The original contributions presented in the study are included in the article/Supplementary material, further inquiries can be directed to the corresponding authors.
