# Supplementary material for: Effects of different physical activity interventions on executive function in older adults with dementia: a systematic review and network meta-analysis
Source: Front Neurol. 2025 Oct 2;16:1643957. doi: 10.3389/fneur.2025.1643957 (PMC12539448; doi:10.3389/fneur.2025.1643957)

Effects of Different Physical Activity Interventions on Executive Function in Older Adults with Dementia: A Systematic Review and Network Meta-Analysis

Contents

Appendix A PRISMA checklist 2

Appendix A1 PRISMA 2020 checklist 2

Appendix A2 PRISMA NMA Checklist 5

Appendix B Search strategy 9

Appendix B1 Initial search strategy 9

Appendix C Publication Bias Test 15

Appendix A PRISMA checklist

Appendix A1 PRISMA 2020 checklist

Section and Topic	Item #	Checklist item	Location where item is reported
**TITLE**			
Title	1	Identify the report as a systematic review.	Page 1
**ABSTRACT**			
Abstract	2	See the PRISMA 2020 for Abstracts checklist.	Page 2-3
**INTRODUCTION**			
Rationale	3	Describe the rationale for the review in the context of existing knowledge.	Page 3-7
Objectives	4	Provide an explicit statement of the objective(s) or question(s) the review addresses.	Page 3-7
**METHODS**			
Eligibility criteria	5	Specify the inclusion and exclusion criteria for the review and how studies were grouped for the syntheses.	Page7-12
Information sources	6	Specify all databases, registers, websites, organisations, reference lists and other sources searched or consulted to identify studies. Specify the date when each source was last searched or consulted.	Page 7-12
Search strategy	7	Present the full search strategies for all databases, registers and websites, including any filters and limits used.	Page 7-12
Selection process	8	Specify the methods used to decide whether a study met the inclusion criteria of the review, including how many reviewers screened each record and each report retrieved, whether they worked independently, and if applicable, details of automation tools used in the process.	Page 9-10
Data collection process	9	Specify the methods used to collect data from reports, including how many reviewers collected data from each report, whether they worked independently, any processes for obtaining or confirming data from study investigators, and if applicable, details of automation tools used in the process.	Page10
Data items	10a	List and define all outcomes for which data were sought. Specify whether all results that were compatible with each outcome domain in each study were sought (e.g. for all measures, time points, analyses), and if not, the methods used to decide which results to collect.	Page 9-10
	10b	List and define all other variables for which data were sought (e.g. participant and intervention characteristics, funding sources). Describe any assumptions made about any missing or unclear information.	Page 9-10
Study risk of bias assessment	11	Specify the methods used to assess risk of bias in the included studies, including details of the tool(s) used, how many reviewers assessed each study and whether they worked independently, and if applicable, details of automation tools used in the process.	Page 11
Effect measures	12	Specify for each outcome the effect measure(s) (e.g. risk ratio, mean difference) used in the synthesis or presentation of results.	Page 11-12
Synthesis methods	13a	Describe the processes used to decide which studies were eligible for each synthesis (e.g. tabulating the study intervention characteristics and comparing against the planned groups for each synthesis (item #5)).	Page 7-12

Section and Topic	Item #	Checklist item	Location where item is reported
	13b	Describe any methods required to prepare the data for presentation or synthesis, such as handling of missing summary statistics, or data conversions.	Page 10-12
	13c	Describe any methods used to tabulate or visually display results of individual studies and syntheses.	Page 11-12
	13d	Describe any methods used to synthesize results and provide a rationale for the choice(s). If meta-analysis was performed, describe the model(s), method(s) to identify the presence and extent of statistical heterogeneity, and software package(s) used.	Page 11-12
	13e	Describe any methods used to explore possible causes of heterogeneity among study results (e.g. subgroup analysis, meta-regression).	Page 11-12
	13f	Describe any sensitivity analyses conducted to assess robustness of the synthesized results.	Page 10-11
Reporting bias assessment	14	Describe any methods used to assess risk of bias due to missing results in a synthesis (arising from reporting biases).	Page 10-11
Certainty assessment	15	Describe any methods used to assess certainty (or confidence) in the body of evidence for an outcome.	Page 11-12
**RESULTS**			
Study selection	16a	Describe the results of the search and selection process, from the number of records identified in the search to the number of studies included in the review, ideally using a flow diagram.	Page 13
	16b	Cite studies that might appear to meet the inclusion criteria, but which were excluded, and explain why they were excluded.	Page 13
Study characteristics	17	Cite each included study and present its characteristics.	Page 13-14
Risk of bias in studies	18	Present assessments of risk of bias for each included study.	Page 14-15
Results of individual studies	19	For all outcomes, present, for each study: (a) summary statistics for each group (where appropriate) and (b) an effect estimate and its precision (e.g. confidence/credible interval), ideally using structured tables or plots.	Page 14-15
Results of syntheses	20a	For each synthesis, briefly summarise the characteristics and risk of bias among contributing studies.	Page 14
	20b	Present results of all statistical syntheses conducted. If meta-analysis was done, present for each the summary estimate and its precision (e.g. confidence/credible interval) and measures of statistical heterogeneity. If comparing groups, describe the direction of the effect.	Page 15-17
	20c	Present results of all investigations of possible causes of heterogeneity among study results.	Page 15-17
	20d	Present results of all sensitivity analyses conducted to assess the robustness of the synthesized results.	Page 15-17
Reporting biases	21	Present assessments of risk of bias due to missing results (arising from reporting biases) for each synthesis assessed.	Page 15-17
Certainty of evidence	22	Present assessments of certainty (or confidence) in the body of evidence for each outcome assessed.	Page 15-17
**DISCUSSION**			

Section and Topic	Item #	Checklist item	Location where item is reported
Discussion	23a	Provide a general interpretation of the results in the context of other evidence.	Page 17-19
	23b	Discuss any limitations of the evidence included in the review.	Page 19-20
	23c	Discuss any limitations of the review processes used.	Page 19-20
	23d	Discuss implications of the results for practice, policy, and future research.	Page 20-21
**OTHER INFORMATION**			
Registration and protocol	24a	Provide registration information for the review, including register name and registration number, or state that the review was not registered.	Page 7
	24b	Indicate where the review protocol can be accessed, or state that a protocol was not prepared.	Page 7
	24c	Describe and explain any amendments to information provided at registration or in the protocol.	Page 7
Support	25	Describe sources of financial or non-financial support for the review, and the role of the funders or sponsors in the review.	Page 23-24
Competing interests	26	Declare any competing interests of review authors.	Page 23-24
Availability of data, code and other materials	27	Report which of the following are publicly available and where they can be found: template data collection forms; data extracted from included studies; data used for all analyses; analytic code; any other materials used in the review.	Page 23-24

Appendix A2 PRISMA NMA Checklist

Section/Topic	Item #	Checklist Item	Reported on Page #
**TITLE**			
Title	1	Identify the report as a systematic review *incorporating a network meta-analysis (or related form of meta-analysis).*	1
**ABSTRACT**			
Structured summary	2	Provide a structured summary including, as applicable:<br>**Background:** main objectives<br>**Methods:** data sources; study eligibility criteria, participants, and interventions; study appraisal; and *synthesis methods, such as network meta-analysis.*<br>**Results:** number of studies and participants identified; summary estimates with corresponding confidence/credible intervals; *treatment rankings may also be discussed. Authors may choose to summarize pairwise comparisons against a chosen treatment included in their analyses for brevity.*<br>**Discussion/Conclusions:** limitations; conclusions and implications of findings.<br>**Other:** primary source of funding; systematic review registration number with registry name.	2-3
**INTRODUCTION**			
Rationale	3	Describe the rationale for the review in the context of what is already known*, including mention of why a network meta-analysis has been conducted.*	4-6
Objectives	4	Provide an explicit statement of questions being addressed, with reference to participants, interventions, comparisons, outcomes, and study design (PICOS).	7
**METHODS**			
Protocol and registration	5	Indicate whether a review protocol exists and if and where it can be accessed (e.g., Web address); and, if available, provide registration information, including registration number.	6-7

Eligibility criteria	6	Specify study characteristics (e.g., PICOS, length of follow-up) and report characteristics (e.g., years considered, language, publication status) used as criteria for eligibility, giving rationale. *Clearly describe eligible treatments included in the treatment network, and note whether any have been clustered or merged into the same node (with justification).*	6-10
Information sources	7	Describe all information sources (e.g., databases with dates of coverage, contact with study authors to identify additional studies) in the search and date last searched.	7
Search	8	Present full electronic search strategy for at least one database, including any limits used, such that it could be repeated.	7-8
Study selection	9	State the process for selecting studies (i.e., screening, eligibility, included in systematic review, and, if applicable, included in the meta-analysis).	7-9
Data collection process	10	Describe method of data extraction from reports (e.g., piloted forms, independently, in duplicate) and any processes for obtaining and confirming data from investigators.	10
Data items	11	List and define all variables for which data were sought (e.g., PICOS, funding sources) and any assumptions and simplifications made.	10-12
**Geometry of the network**	**S1**	Describe methods used to explore the geometry of the treatment network under study and potential biases related to it. This should include how the evidence base has been graphically summarized for presentation, and what characteristics were compiled and used to describe the evidence base to readers.	11-12
Risk of bias within individual studies	12	Describe methods used for assessing risk of bias of individual studies (including specification of whether this was done at the study or outcome level), and how this information is to be used in any data synthesis.	9-11
Summary measures	13	State the principal summary measures (e.g., risk ratio, difference in means). *Also describe the use of additional summary measures assessed, such as treatment rankings and surface under the cumulative ranking curve (SUCRA) values, as well as modified approaches used to present summary findings from meta-analyses.*	19-11
Planned methods of analysis	14	Describe the methods of handling data and combining results of studies for each network meta-analysis. This should include, but not be limited to:<br>• *Handling of multi-arm trials;*<br>• *Selection of variance structure;*<br>• *Selection of prior distributions in Bayesian analyses; and*<br>• *Assessment of model fit.*	11-12

**Assessment of Inconsistency**	**S2**	Describe the statistical methods used to evaluate the agreement of direct and indirect evidence in the treatment network(s) studied. Describe efforts taken to address its presence when found.	10-12
Risk of bias across studies	15	Specify any assessment of risk of bias that may affect the cumulative evidence (e.g., publication bias, selective reporting within studies).	10-12
Additional analyses	16	Describe methods of additional analyses if done, indicating which were pre-specified. This may include, but not be limited to, the following:<br>• Sensitivity or subgroup analyses;<br>• Meta-regression analyses;<br>• *Alternative formulations of the treatment network; and*<br>• *Use of alternative prior distributions for Bayesian analyses (if applicable).*	11-12
**RESULTS**			
Study selection	17	Give numbers of studies screened, assessed for eligibility, and included in the review, with reasons for exclusions at each stage, ideally with a flow diagram.	12-13
**Presentation of network structure**	**S3**	Provide a network graph of the included studies to enable visualization of the geometry of the treatment network.	13-14
**Summary of network geometry**	**S4**	Provide a brief overview of characteristics of the treatment network. This may include commentary on the abundance of trials and randomized patients for the different interventions and pairwise comparisons in the network, gaps of evidence in the treatment network, and potential biases reflected by the network structure.	14-15
Study characteristics	18	For each study, present characteristics for which data were extracted (e.g., study size, PICOS, follow-up period) and provide the citations.	14-15
Risk of bias within studies	19	Present data on risk of bias of each study and, if available, any outcome level assessment.	14
Results of individual studies	20	For all outcomes considered (benefits or harms), present, for each study: 1) simple summary data for each intervention group, and 2) effect estimates and confidence intervals. *Modified approaches may be needed to deal with information from larger networks.*	13-15
Synthesis of results	21	Present results of each meta-analysis done, including confidence/credible intervals. *In larger networks, authors may focus on comparisons versus a*	14-16

		particular comparator (e.g. placebo or standard care), with full findings presented in an appendix. League tables and forest plots may be considered to summarize pairwise comparisons. If additional summary measures were explored (such as treatment rankings), these should also be presented.	
**Exploration for inconsistency**	**S5**	Describe results from investigations of inconsistency. This may include such information as measures of model fit to compare consistency and inconsistency models, *P* values from statistical tests, or summary of inconsistency estimates from different parts of the treatment network.	15-16
Risk of bias across studies	22	Present results of any assessment of risk of bias across studies for the evidence base being studied.	15-16
Results of additional analyses	23	Give results of additional analyses, if done (e.g., sensitivity or subgroup analyses, meta-regression analyses*, alternative network geometries studied, alternative choice of prior distributions for Bayesian analyses,* and so forth).	14-16
**DISCUSSION**			
Summary of evidence	24	Summarize the main findings, including the strength of evidence for each main outcome; consider their relevance to key groups (e.g., healthcare providers, users, and policy-makers).	16-19
Limitations	25	Discuss limitations at study and outcome level (e.g., risk of bias), and at review level (e.g., incomplete retrieval of identified research, reporting bias). *Comment on the validity of the assumptions, such as transitivity and consistency. Comment on any concerns regarding network geometry (e.g., avoidance of certain comparisons).*	19-20
Conclusions	26	Provide a general interpretation of the results in the context of other evidence, and implications for future research.	20-21
**FUNDING**			
Funding	27	Describe sources of funding for the systematic review and other support (e.g., supply of data); role of funders for the systematic review. This should also include information regarding whether funding has been received from manufacturers of treatments in the network and/or whether some of the authors are content experts with professional conflicts of interest that could affect use of treatments in the network.	21-22

Appendix B Search strategy

Appendix B1 Initial search strategy

Database	Search strategy	amount
	**PubMed**	
#1	(((((((((((Physical activity[MeSH Terms]) OR ([Physical activity[Title/Abstract])) OR (Aerobic activity[Title/Abstract])) OR (Recreation activities[Title/Abstract])) OR ([Physical exercise[Title/Abstract])) OR (Free-time activities[Title/Abstract])) OR (Leisure-time physical activity[Title/Abstract])) OR (Well-being activities[Title/Abstract])) OR (exercise[Title/Abstract]))	942,103 results
#2	((((((((Dementia[MeSH Terms]) OR (Dementia[Title/Abstract])) OR (Neurocognitive Disorders[MeSH Terms])) OR (Cognitive Dysfunction[Title/Abstract])) OR (Senile Dementia[Title/Abstract])) OR (Alzheimer's Disease[Title/Abstract]) OR (Vascular Dementia[Title/Abstract])) OR (Lewy Body Dementia[Title/Abstract])) OR (Frontotemporal Dementia[Title/Abstract]))	459,865 results
#3	((((((((Older Adults[MeSH Terms]) OR (Older Adults[Title/Abstract])) OR (Aged[Title/Abstract])) OR (Aged, 80 and over[Title/Abstract])) OR (aging population[Title/Abstract])) OR (Octogenarians[Title/Abstract])) OR (elderly[Title/Abstract])) OR (Geriatric population[Title/Abstract]))	4,347,934 results
#4	((((executive function[MeSH Terms] OR (executive function)) OR (planning)) OR (inhibitory control)) OR (cognitive flexibility)) OR (working memory))	906,513 results
#5	((((Randomized controlled trial[MeSH Terms] OR (Randomized controlled trial)) OR (controlled	2,020,366

	**clinical trial)) OR (randomized)) OR (placebo)) OR (randomly))**	results
#6	#1 AND #2 AND#3 AND #4 And #5	503 results

Embase		
#1	**'Physical activity'/exp OR 'Physical activity' OR 'Aerobic activity' OR 'Recreation activities' OR 'Physical exercise' OR 'Free-time activities' OR 'Leisure-time physical activities' OR 'Well-being activities' OR 'exercise'**	1,276,727
#2	'dementia'/exp OR 'dementia' OR 'neurocognitive disorder' OR 'cognitive dysfunction' OR 'senile dementia' OR 'alzheimer disease' OR 'vascular dementia' OR 'lewy body dementia' OR 'frontotemporal dementia'	584,927
#3	**'Older Adults'**/exp OR 'Older Adults' OR 'aged' OR 'aged 80 and over' OR 'octogenarians' OR 'aging population' OR 'elderly' OR 'geriatric population'	6,621,508
#4	**'executive function'/exp OR 'executive function' OR 'planning' OR 'inhibitory control' OR 'cognitive flexibility' OR 'working memory'**	915,834
#5	**'randomized controlled trial'/exp OR 'randomized controlled trial' OR 'randomised controlled trial' OR 'controlled clinical trial' OR 'RCT' OR 'randomly allocated' OR 'placebo-controlled' OR 'randomized' OR 'placebo' OR 'clinical trial'**	2,957,138
#6	#1 And #2 And #3 And #4 And #5	528

Web of Science		
#1	**(((((((TS=(Physical activity)) OR TS=(Recreation activities)) OR TS=(Aerobic activity)) OR TS=(free-time activities)) OR TS=(Physical exercise)) OR TS=(leisure-time physical activity)) OR TS=(well-being activities)) OR TS=(exercise) and Preprint Citation Index (Exclude – Database)**	1,912,993
#2	**(((((((TS=(dementia)) OR TS=(neurocognitive disorder)) OR TS=(cognitive dysfunction)) OR TS=(senile dementia)) OR TS=(alzheimer disease)) OR TS=(vascular dementia)) OR TS=(lewy body dementia)) OR TS=(frontotemporal dementia) and Preprint Citation Index (Exclude – Database)**	590,519
#3	**((((((TS=(Older Adults)) OR TS=(aged)) OR TS=(aged 80 and over)) OR TS=(octogenarians)) OR TS=(aging population)) OR TS=(elderly)) OR TS=(geriatric population)and Preprint Citation Index (Exclude – Database)**	11,207,8<br>20
#4	**TS=("executive function" OR "planning" OR "inhibitory control" OR "cognitive flexibility" OR "working memory")**	1,204,95<br>1
#5	**TS=("randomized controlled trial" OR "randomised controlled trial" OR "controlled clinical trial" OR "RCT" OR "randomly allocated" OR "placebo-controlled") OR TI=("randomized" OR "placebo" OR "clinical trial")**	737,243
#6	#1 AND #2 And #3 And #4 And #5	412

Cochrane		
#1	MeSH descriptor: [Physical activity] explode all trees	68248
#2	(Physical activity):ti,ab,kw OR (Recreation activities):ti,ab,kw OR (Aerobic activity):ti,ab,kw OR (Physical exercise):ti,ab,kw OR (free-time activities):ti,ab,kw	93288
#3	(leisure-time physical activity):ti,ab,kw OR (well-being activities):ti,ab,kw OR (exercise):ti,ab,kw	143367
#4	#1 OR #2 OR #3	180028
#5	MeSH descriptor: [dementia] explode all trees	31919
#6	(dementia):ti,ab,kw OR (neurocognitive disorder):ti,ab,kw OR (cognitive dysfunction):ti,ab,kw OR (senile dementia):ti,ab,kw OR (alzheimer disease):ti,ab,kw	35134
#7	(vascular dementia):ti,ab,kw OR (lewy body dementia):ti,ab,kw OR (frontotemporal dementia):ti,ab,kw	2513
#8	#5 OR #6 OR #7	43471
#9	MeSH descriptor: [Older Adults] explode all trees	31450
#10	(Older Adults):ti,ab,kw OR (aged 80 and over):ti,ab,kw OR (aged):ti,ab,kw OR (aging population):ti,ab,kw OR (elderly):ti,ab,kw	707832

#11	(octogenarians):ti,ab,kw OR (geriatric population):ti,ab,kw	1958
#12	#9 OR #10 OR #11	710563
#13	MeSH descriptor: [executive function] explode all trees	9102
#14	(executive function):ti,ab,kw OR (planning):ti,ab,kw OR (inhibitory control):ti,ab,kw OR (cognitive flexibility):ti,ab,kw OR (working memory):ti,ab,kw	41243
#15	#13 OR #14	41775
#16	MeSH descriptor: [randomized controlled trial] explode all trees	1132890
#17	(randomized controlled trial):ti,ab,kw OR (randomised controlled trial):ti,ab,kw OR (controlled clinical trial):ti,ab,kw OR (RCT):ti,ab,kw OR (randomly allocated):ti,ab,kw	874279
#18	(randomized):ti,ab,kw OR (placebo):ti,ab,kw OR (clinical trial):ti,ab,kw OR (placebo-controlled):ti,ab,kw	1418457
#19	#16 or #17 or #18	1510465
#17	#4 and #8 and #12 and #15 and #19	810

Appendix C Risk of bias assessment of included studies

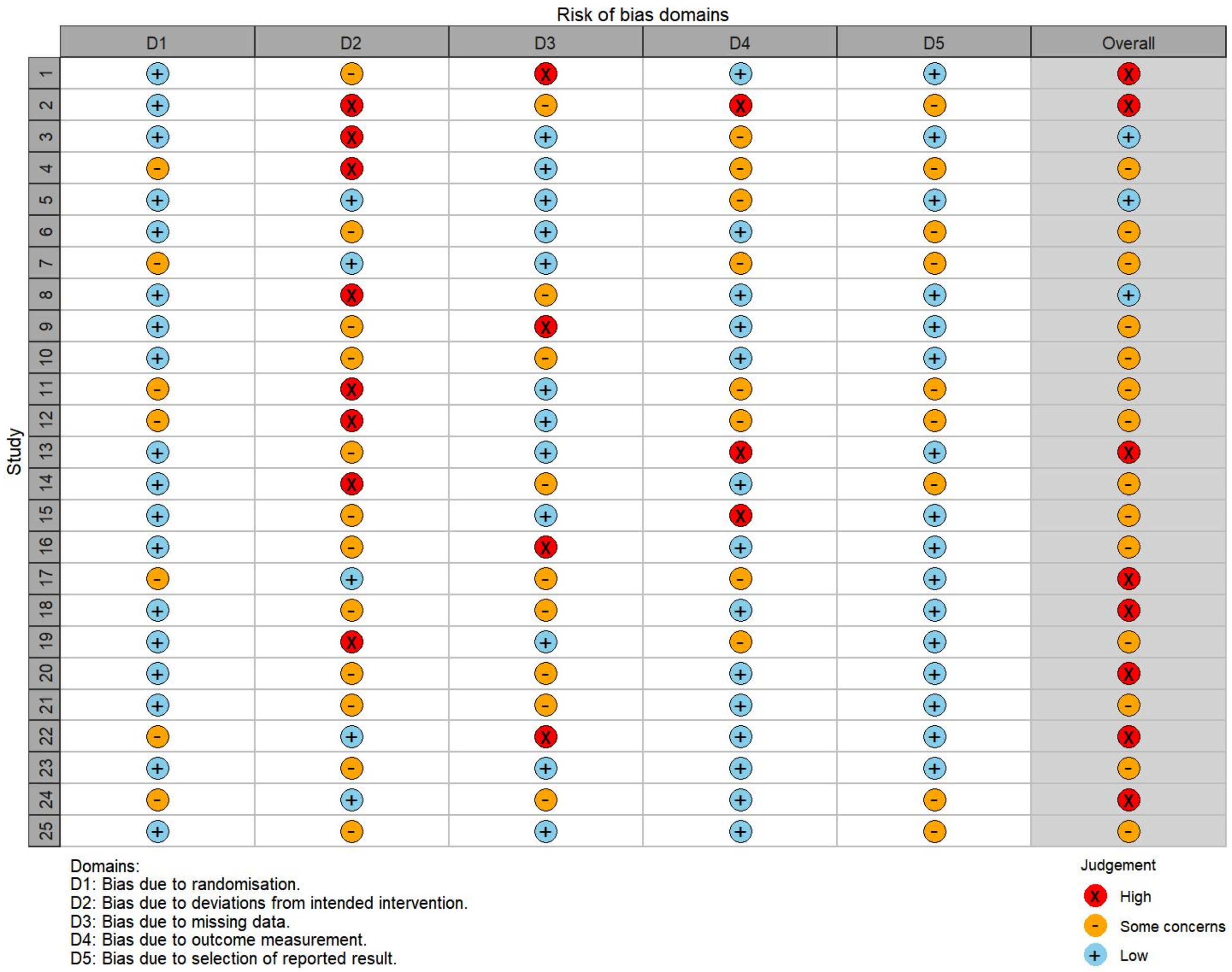

Supplement: Supplementary file 1 [file Data_Sheet_1.PDF]
